# Seeing Aging Anew: Using Arts-Based Teaching to Enrich Interprofessional Geriatrics Education

**DOI:** 10.1093/geroni/igaf122.1359

**Published:** 2025-12-31

**Authors:** Mariah Robertson, Kelseanne Breder

**Affiliations:** Johns Hopkins School of Medicine, COLUMBIA, Maryland, United States; New York University, New York, New York, United States

## Abstract

Arts and humanities-based pedagogy encourages a human-centered approach to learning that is valuable to all health professions education (HPE). This type of teaching promotes empathy, tolerance of ambiguity, capacity to wonder, observation and communication skills, and assists with dialogue across differences. These are all priority skills for people who serve older adults across the educational continuum. Arts-based education can also help to reframe aging, building interest and investment in geriatrics among non-geriatrics trained clinicians. In this presentation, we review the uses of arts-based teaching implemented across a variety of educational settings including the home and nursing home for interprofessional learners from paramedicine, medicine, nursing and public health. We will specifically highlight the core aspects of two arts-based curricular innovations focused on geriatrics education and will assist participants in identifying opportunities to use components of these curricula in their own HPE.

